# Temporal Frame of Immune Cell Infiltration during Heart Failure Establishment: Lessons from Animal Models

**DOI:** 10.3390/ijms19123719

**Published:** 2018-11-22

**Authors:** David Brenes-Castro, Elena C. Castillo, Eduardo Vázquez-Garza, Guillermo Torre-Amione, Gerardo García-Rivas

**Affiliations:** 1 Tecnologico de Monterrey, Escuela de Medicina y Ciencias de la Salud, Cátedra de Cardiología y Medicina Vascular, Monterrey, Nuevo León 64849, Mexico; dbrenesc@gmail.com (D.B.-C.); ecgonzalez@itesm.mx (E.C.C.); EduardoVzGz@itesm.mx (E.V.-G.); gtorre@tecsalud.mx (G.T.-A.); 2 Tecnologico de Monterrey, Hospital Zambrano Hellion, TecSalud, Centro de Investigación Biomédica, San Pedro Garza García, Nuevo León 66278, Mexico; 3 Methodist DeBakey Heart & Vascular Center, The Methodist Research Institute, Weill Cornell Medical College, Houston, TX 77030, USA

**Keywords:** heart failure, inflammation, animal models, pressure overload

## Abstract

Heart failure (HF) is a cardiovascular syndrome characterized by maladaptive changes with an underlying inflammatory mediated pathogenesis. Nevertheless, current therapy is aimed at the heart workload and neurohormonal axis; thus, prognosis remains poor. To continue improving treatment, we rely on murine models for a better understanding of HF pathophysiology. Among them, pressure overload HF (PO-HF) animal models are a common strategy. Development of PO-HF is characterized by monocyte infiltration, which orchestrates a cascade of events leading to sustained inflammation and maladaptive changes. Here, we divide the PO-HF model progression into four phases and describe the inflammatory, structural, and gene expression profiles. This division is relevant due to its similarities with clinical hypertensive heart disease progression to HF. Evidence shows improvement in hemodynamic and other local parameters by altering the inflammatory response in a specific immune response at a specific point of time. Thus, it is relevant to focus on the time-dependent immune response interaction in order to provide more effective therapy. This review summarizes the pathogenesis of PO-HF murine models, highlighting the inflammatory events in a time frame view. By this approach, we expect to provide researchers with a better understanding of the intertwining time-dependent events that occur in PO-HF.

## 1. Introduction

Heart failure (HF) is a cardiovascular syndrome characterized by a structural and/or functional cardiac abnormality, resulting in reduced cardiac output and/or elevated intracardiac pressures [1]. Clinically, HF is classified based on its ejection fraction (EF) as reduced if <40% (heart failure reduced ejection fraction; HFrEF), moderately reduced between 40–49%, and preserved if >50% (heart failure preserved ejection fraction; HFpEF) [1].

The exact prevalence of HF is difficult to assess due to its dependence on the definition applied as well as the diagnostic methods and their availability (e.g., BNP (brain natriuretic peptide) and echocardiography). Furthermore, the continuously increasing lifespan of the general population, aided by the elongation of cardiac patients’ lives, is leading to an increase in HF prevalence with a positive correlation with age [2,3]. Nevertheless, despite improvements in therapy, the current prognosis remains poor, with a high 12-month and 5-year all-cause mortality, mostly by cardiovascular causes [1,4,5,6].

Even though persistent inflammation and extracellular matrix remodeling are recognized as central in the pathogenesis of HF [7,8], the current therapy offered to HF patients is aimed at the heart workload (e.g., beta blockers) and neurohormonal axis (e.g., Angiotensin-converting enzyme inhibitors (ACEi), both with hypotension and bradycardia as their main limiting factors [9]. The role of inflammation in HF was first described in 1990 by Levine et al., who established a correlation with the cytokine tumor necrosis factor α (TNFα), which was further evolved in the “Cytokine Hypothesis of Heart Failure” [10,11], in which a precipitating event could trigger elaboration of proinflammatory cytokines, accelerating the progression of HF [10,12]. Among the deleterious effects of proinflammatory cytokines are the promotion of necrosis, apoptosis, hypertrophic response, left ventricle dilation, and negative inotropic effects [13,14]. Now, many aspects of its pathogenesis can be explained by the known biological effects of cytokines, and the pattern of expression of cytokines is related to the progression of HF [13,14,15].

Following this line of thought and the limitations of the current therapy, there have been a variety of inflammatory mediators that researchers have attempted to use as therapeutic targets, among them TNFα and, more recently, interleukin(IL)-1β [13,16]. The former was targeted with the soluble TNF receptor, Etanercept [17], and TNFα monoclonal antibody, Infliximab [18], but unfortunately results were not promising. However, (IL)-1β targeted with Canakinumab was indeed able to reduce inflammatory biomarkers and cardiovascular events in patients with coronary disease [19]. Furthermore, new evidence obtained with HF animal models using alternative approaches could still highlight the potential for an inflammatory therapeutic strategy. Of note, even though many of these results come from ischemic HF models, these findings are relevant because of some key shared features as compared with pressure overload HF (PO-HF) or other etiologies [13,20].

The purpose of this review is to describe the pathogenesis of PO-HF by highlighting the inflammatory events and their continuous changes in a novel and unique time frame view. Although previous reviews have also focused on the inflammatory role in HF pathogenesis, they described inflammation as separate arms, without providing a clear time-dependent interaction of the inflammatory cascade as a continuum of maladaptive changes that characterize the pathogenesis of PO-HF. Through our approach, we expect to provide researchers with a better understanding of the intertwining time-dependent events that occur in PO-HF.

## 2. Heart Failure Animal Models

Currently, our understanding of HF is not yet sufficient to precisely discriminate etiologies according to their pathogenesis [21]. Resembling different etiologies, HF animal models allow us to analyze the physiological effects of cardiac dysfunction in the overall HF phenotype [8,21]. Even though there are several limitations to using murine models, such as the endpoints used in clinical studies (e.g., hospital admissions and quality of life), structural heart differences, and patients’ multiple comorbidities, among others, we still rely on animal models to understand the pathophysiology of HF [21]. Reviewing all of the murine HF models is beyond the scope of this article. Here, we present a list of the common HF murine models with particular focus on the thoracic aortic constriction (TAC) and angiotensin II (ATII) infusion models due to their relevance and resemblance to each other. In this review, pressure overload (PO) model will be used indistinctly either for TAC or ATII infusion, unless otherwise specified. As it can be observed in Table 1, these two models only differ in the EF. Even though the TAC model creates a gradually PO increase, there is evidence that afterload increases just hours after the procedure [22]; given the procedural variability (i.e., grade of initial constriction), timing of surgery, and the invariable progression to HFrEF, it is not considered to be a true HFpEF model [21]. On the other hand, the changes observed in the ATII infusion model are both strain- and dose-dependent, which gives this technique the capacity to manipulate the model at the experimental design in order to make it more alike to a HFpEF model [23,24].

In this review, HF murine models are, by their etiology, either ischemic or non-ischemic due to more clinical relevance by this approach. In the former group, the more common technique is the surgical clipping of coronary arteries, typically the left descending anterior. In the non-ischemic group, the more common techniques are thoracic aortic constriction, typically the transverse aortic constriction (TAC), ATII infusion, and aldosterone-infused nephrectomized mice, all three of which resemble a PO etiology. Other methods of non-ischemic HF models are metabolic models (e.g., obesity and diabetes), aging models (e.g., mouse spontaneous senescence), autoimmune models (e.g., post-myocarditis HF), toxin-induced models (e.g., isoproterenol, ethanol, or doxorubicin), and genetic models of cardiomyopathies (CMP) [14,21,25,28,29]. It is important to note that even though there are different methods (i.e., etiologies), HF models share a fibrotic response and remodeling changes, each model with different degrees or specific characteristics. However, there are some key differences between ischemic and non-ischemic etiologies that will be reviewed next. For this review, the PO model will be used indistinctly either for TAC or ATII infusion, unless otherwise specified.

### 2.1. Ischemic vs. Non-Ischemic (Pressure Overload Model)

**Figure 1:**
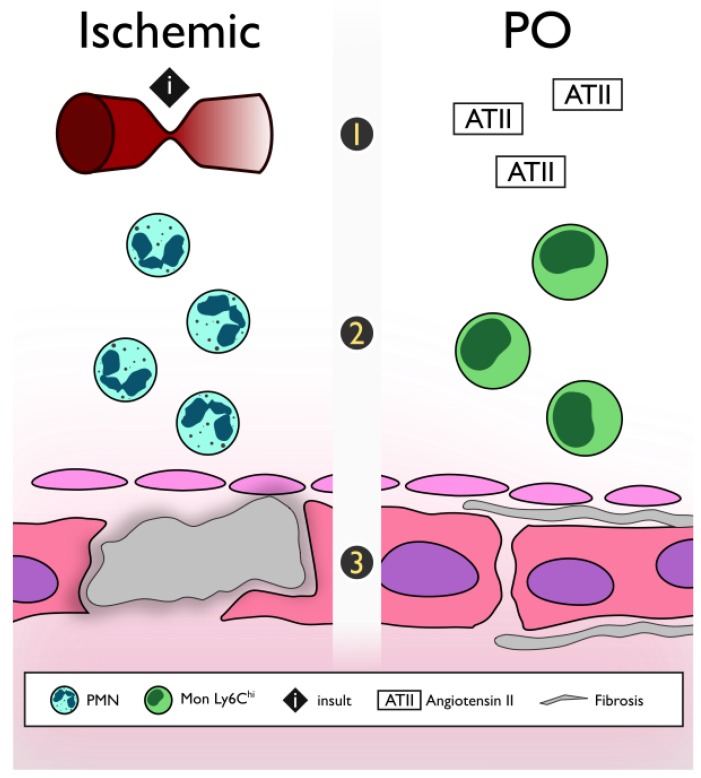
Key differences between the ischemic and PO-HF murine model. (**1**) Type of insult, (**2**) First to arrive: type of cellular infiltrate, and (**3**) type of fibrosis. Mon Ly6C^hi^: inflammatory monocytes; PMN: Polymorphonuclear neutrophils; PO: Pressure overload.

There are three main differences between these etiologies (Figure 1).

#### 2.1.1. Acute Insult vs. Progressive

On the one hand, it is well known that after an acute insult (such as ischemia in the post-myocardial infarction HF), necrosis of the tissue results, which is itself a powerful inflammatory stimulus to trigger an inflammatory cascade. On the other hand, the non-ischemic model despite no acute nor direct damage is induced to the myocardium, there is sufficient evidence that specifically points to ATII as the critical mediator of inflammation in the PO model, which may directly stimulate inflammatory pathways through generation of reactive oxygen species and activation of the nuclear factor κB (NF-κB) pathway [27,30,31,32,33]. Additionally, pressure overload may induce alterations in the cardiac extracellular matrix through activation of matrix metalloproteinases (MMPs). Hence, matrix fragments may serve as damage-associated molecules patterns (DAMPs) that activate Toll-Like Receptors (TLRs), triggering inflammation through the NF-κB pathway [34,35,36].

#### 2.1.2. First to Arrive

Following ischemia, neutrophils are the first inflammatory cell to arrive in the myocardium followed by monocytes to further exacerbate the inflammatory response. In contrast, in non-ischemic models, monocytes are the first immune cell to arrive and are responsible for orchestrating the inflammatory cascade [37,38,39]. It has been shown that ATII promotes the monocytes chemotaxis from the bone marrow and spleen [40,41]

#### 2.1.3. Beneficial vs. Pathological Fibrosis

Even though it is well known that excessive fibrosis is detrimental to the mechanical properties and function of the heart, after necrosis induced by ischemia, tissue healing mediated by a fibrotic response is necessary for a proper recovery. This has been called reparative or replacement fibrosis, and it is characterized by a collagen type III predominance [42,43]. In contrast, although the activation of transforming growth factor-β TGF-β signaling pathways suppresses inflammation in the non-ischemic heart, this results in pure maladaptive fibrotic remodeling due to the absence of significant cardiomyocyte loss. This fibrotic process is called reactive fibrosis with predominance collagen type 1 [38,44].

### 2.2. Pressure Overload

**Figure 2:**
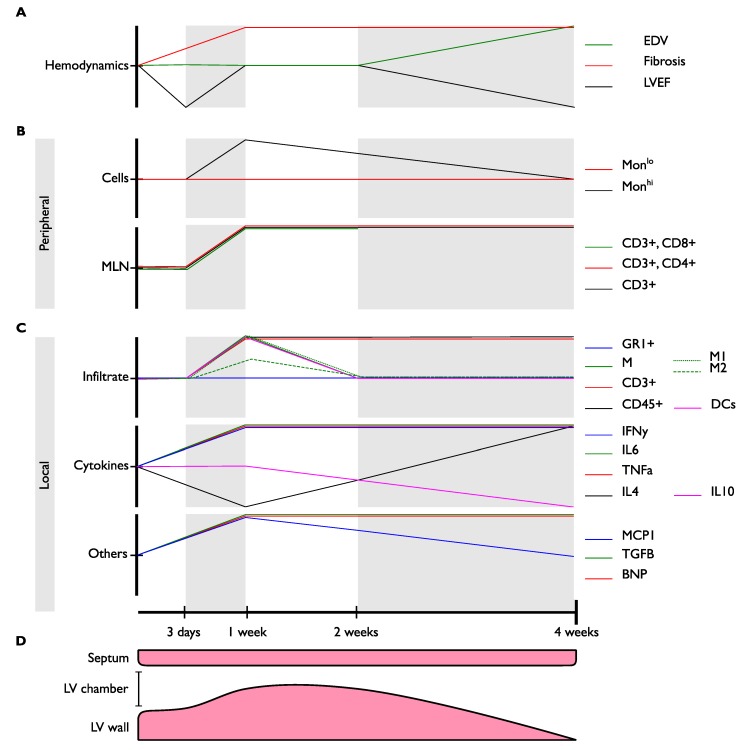
Graphical representation of changes through time in PO-HF. This figure is divided vertically in four panels (**A** to **D**) representing different groups of parameters evaluated; each panel has at its right end, the color code that represents each color line; and along the X-axis at the bottom, a timeline can be observed representing critical time frames for the PO-HF model. The graphical values plotted represent significant changes for increased or decreased values compared with their respective control group at the same moment of measurement. (**A**) Hemodynamic parameters. (**B**) Peripheral cell populations. (**C**) Heart tissue measurements. (**D**) Thickness of LV wall and dimensions of LV chamber. X-axis represents time. Y-axis is divided into a different set of measured parameters. Values represent significant changes for increased or decreased values compared with control groups. MLN: Mediastinal lymph nodes; EDV: End diastolic volume; LVEF: Left ventricle ejection fraction; Mon: Monocytes; M: Macrophages; M1: Pro-inflammatory macrophages; M2: Anti-inflammatory macrophages; DCs: Classical dendritic cells; BNP: Brain natriuretic peptide.

The development of PO-HF is characterized by structural and metabolic changes within the myocardium, including cardiomyocyte hypertrophy, interstitial/reactive fibrosis, inflammation, and a switch toward a fetal-like metabolic profile, among others [45,46,47]. In the PO-HF model, progression of the pathogenesis can be represented in a timeline divided into four phases marked by their hemodynamic changes (Figure 2): (1) Acute decompensated phase (ADP), (2) compensated phase (CP), (3) DP and HF, and (4) congestive HF (CHF) and end-stage HF [40,47,48,49]. Here, we describe each phase and correlate hemodynamic and remodeling changes with the inflammatory characteristics at that moment in time. A more detailed description of every phase is shown in Table 2.

## 3. Understanding the Different Roles of Immune Response

### 3.1. T and B Cells

Different strategies have been used to study the role of lymphocytes in contributing to the pathogenesis for sustained inflammation and adverse remodeling in the PO model. A recent work by Cordero-Reyes et al. [50] used SCID mice (i.e., lacking B and T cells), mice treated with anti-CD22 (i.e., CD22 mice lacking B cells), and nude mice (i.e., lacking T cells) in an ATII infusion PO-HF murine model to evaluate the separate roles of the lymphocytes. Even though the hypertensive response was equal among all groups, they did find similar detrimental results in heart remodeling and fibrotic response in both the wild type (WT) and nude CMP groups compared to the SCID and CD22 CMP groups, in which those changes were less evident. These findings can be partially attributed to a more relevant role of the B cells over the T cells in those parameters.

Furthermore, following B cell reconstitution in the SCID mice, hypertrophy, collagen deposition, and BNP expression were significantly increased compared to the SCID CMP group. These findings further suggest the influential role of the B lymphocytes. Regarding heart cytokine gene expression of IL-1β, IL-6, and TNFα, all were increased in the WT and SCID+B cell CMP groups compared with the CTL group; IL-1β and IL-6 were also increased in the T cell-deficient group. Of note, the CD22 CMP group was the only group without decreased expression of IL-10. Also, staining for IgG3 was positive in the WT, nude, and SCID+B cell CMP groups. These results, obtained after reconstitution of B cells in the SCID group along with its correlation with IgG3 staining, show the key role of B cells in the pathogenesis of PO-HF and further support their antibody production role, which has already been discovered in advanced HF patients [50,52,53].

Furthermore, there have been some studies that correlated B cells with the mobilization of monocytes, likely via CCL7 production of the former [53]. Thus, further research on this relationship in PO-HF models is needed. Nevertheless, it is well known that B cells modulate the T cell response by acting as antigen presenting cells (APC), meaning that by modulating B cell activity, they also affect T cell response and possible monocyte infiltration. Following this line of thought, other studies addressed not only the role of T cells, but also this APC activity. One of these works, by Laroumanie et al. [49], used four different methods in TAC mice models of PO-HF. Among their results for RAG2KO mice (deficient with mature T and B cells), they discovered a recovery of reduction of the FS, prevention of chamber dilation, reduction of BNP expression, and reduced fibrotic response associated with less Ma infiltration; all of these were reversed and comparable to WT after the reconstitution of T cells. These adverse parameters were also similar to WT in T cell CD8KO mice, whereas they were either preserved or reduced in MHCII KO (T cell CD4 deficient) mice. Furthermore, in OTII mice (APC deficient T-cells due to restriction to a specific antigen), FS was preserved, cardiac dilation was prevented, and fibrosis was reduced, thus demonstrating the key pathogenic role of CD4^+^ over CD8^+^ T cells along with the significant importance of their interaction with APC cells.

The latter was also corroborated by Kalliourdis et al. [51] by blocking the T cell co-stimulation with DCs, B cells, and macrophages using abatacept. Through this approach, the FS and EF were significantly preserved during the first, third, and fourth weeks after TAC along with a reduction in BNP expression and fibrotic response. Similar beneficial hemodynamic results of FS and EF were also observed when abatacept was administered during the second week instead of at the beginning of the model. These results were correlated with a reduction in T cells during the fourth week and reduced expression of T cell co-stimulation markers, such as AIF-1 (allograft inflammatory factor 1) and CD25^+^ (regulatory T cells). These findings corroborate the relevance of T cell co-stimulation along the pathogenesis timeline.

Additionally, Nevers et al. [47] found that T cell recruitment is also an essential step in the pathogenesis of heart remodeling. Using TCRα -/- (T cell receptor alpha knockout) mice in a TAC model of PO-HF, they confirmed an LV free of recruited T cells during the fourth week without changes in ICAM expression associated with standard LV dimensions, preserved systolic function (FS and SV), decreased BNP expression, and fibrotic response. Moreover, when T cells were depleted at 48 h post TAC using anti-CD3 antibodies, they also discovered preserved FS and decreased fibrotic response during the fourth week.

### 3.2. Mononuclear Phagocytic Cells: Monocytes, Macrophages, and Dendritic Cells

As previously reported by Patel et al. [40], depletion of all mononuclear phagocytes (MnPs; i.e., peripheral monocytes and DCs) via Fas-induced apoptosis with the drug, AP20187, in MaFIA mice during the second week after TAC was not able to alter the progression of LV dilation, systolic dysfunction, and cardiac remodeling, measured as LV hypertrophy and fibrosis. These results might suggest that the pathophysiological role of MnPs does not affect the pathogenesis after the CP. The following year, using selective CCR2 antagonism with the drug, RS-504393, on the third day after TAC suppressed the infiltration of M1 CCR2+ during the first week after TAC, even though circulating Ly6C^hi^ CCR2^+^ population and expression of adhesion molecules (ICAM-1, VCAM-1) were not affected. This was associated with abrogation of the pathological expansion of total CD3^+^, CD4^+^, and CD8^+^ T cells at MLN, a reduction in BNP expression, and a smaller cardiomyocyte area. Results from the fourth week showed attenuation of LV dilation and systolic dysfunction, a reduction in interstitial fibrosis, and sustainment of the decreased BNP expression in the heart.

Using a second approach, with monoclonal antibody, MC21, to deplete circulating Ly6C^hi^ CCR2+ monocytes during the third to fifth day post-TAC, they observed a reduction in interstitial fibrosis during the fourth week. The latter is associated with suppression of the up-regulation of collagen I, collagen III, collagen 4α3, fibronectin, and vimentin expression in the heart. These findings were associated with better EF (but not with changes in the EDV), a significant reduction in the cardiomyocyte area, and a significant reduction in the T cell population expansion in the heart and MLN [54]. With these results, the authors concluded that first, the initial expansion of heart macrophages is dependent on circulating Ly6C^hi^ monocytes by CCR2/CCL2 interaction. Second, the MnPs role in pathogenesis is only relevant in the initial two weeks after PO, corresponding to the time frame with increased peripheral and local monocytes and the establishment of the CP of PO-HF. Third, MnPs play a crucial role by interacting with other immune cell subsets, such as T-cells [48]. Moreover, it is important to highlight that the role of cytokines produced by heart Ma (e.g., IL-1β) has been correlated with other non-structural abnormalities, such as arrhythmogenesis, which is recognized as an important comorbidity and cause of sudden cardiac death in HF patients [1,55].

## 4. The Inflammatory Pathway and Pathogenesis of the Pressure Overload Heart Failure Model

**Figure 3:**
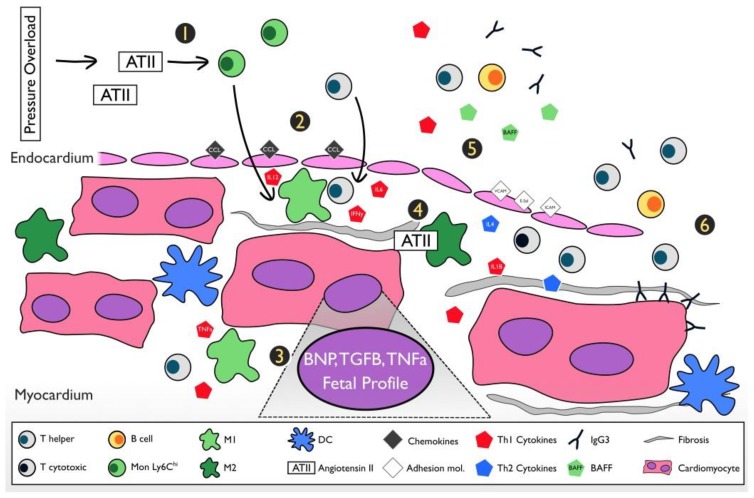
Summary of the PO-HF murine model. **1**) Progressive PO stimulates the release of ATII. ATII stimulates mobilization of Ly6C_hi_ monocytes from the bone marrow and spleen [30,38,40,41]. ATII and mechanical stress stimulate the expression of chemokines promoting infiltration of Ly6C^hi^ Mon following its differentiation into M1 [37,56]. **2**) Pro-inflammatory cytokines expressed in the heart (e.g., TNFα) as well as released from M1 and chemokines further stimulates mobilization and infiltration of CD4^+^ T cells [38,40,47,48]. M1 produce IL-12, which induces IFN-ɣ by T cells that activate macrophages that produce MCP1, creating a positive feedback of monocyte infiltration, M1 activation, and T cell infiltration and activation [54]. **3**) Mechanical stress induces expression of structural genes that lead to a hypertrophic response. **4**) ATII stimulates M2 production of TGFβ, leading to fibroblast stimulation and a reactive fibrotic response [37,39,57]. However, it is still not clear the process of transition from M1 to M2 [58]. Then, macrophage population starts to decline progressively, which correlates with a reduced number of circulating monocytes [47,48], marking the beginning of the transition from an innate to an adaptive response. **5**) Expansion of T cells in the heart and expression of leukocyte adhesion molecules, VCAM1, E-Sel, and ICAM1 [47]. Increased IL-4 and BAFF suggests the participation of B cells, key modulators of the T cell response [49,50,51]. Structurally it starts a progressive increase of EDV [48,50]. **6**) The second peak of DCs is observed and the predominance of the CD4^+^ T cell subset and deposition of IgG3 suggests that the sustained inflammatory response belongs to the adaptive immune response with both cellular and humoral participation [49,50].

Figure 3 shows a summary of key inflammatory events occurring in the progression of the PO-HF model.

## 5. Findings in Animal Models and Their Correlation with Human Heart Failure

### 5.1. Transition from Hypertension to Heart Failure

In clinical practice, most long-standing HTN, unless interrupted (i.e., treated or complicated by an event, such as a myocardial infarction), eventually leads to HF after a series of poorly characterized maladaptive changes [59,60]. Among these changes, LV diastolic dysfunction is almost always the first manifestation of heart compromise; although commonly asymptomatic, if sustained, it progresses to symptomatic decompensation followed by HFpEF [61]. Thus, HTN is the most common associated factor of diastolic dysfunction, followed by HFpEF [56]. Altogether, etiology and cardiac dysfunction encompass a clinical entity known as hypertensive heart disease (HHD). A seven-pathway pathogenesis was proposed by Drazner for the development of HHD [62], in which the route followed depended on factors, like BP severity, comorbidities (e.g., diabetes mellitus, obesity), genetic influences, race, and events, such as myocardial infarction. This pathogenic variability results in a broad clinical spectrum that can lead to either HFrEF or HFpEF with dilated CMP or concentric hypertrophy, respectively.

Furthermore, clinical HHD can be classified into four progressive categories [63]: Degree I, isolated LV diastolic dysfunction with no LV hypertrophy; Degree II, LV diastolic dysfunction with concentric hypertrophy; Degree III, clinical HF (dyspnea and pulmonary edema with preserved EF); and Degree IV, dilated cardiomyopathy with HFrEF. Of note, this proposed hemodynamic progression for HHD shares some similarities with the PO-HF model that will be briefly discussed later in this section (Figure 2; Table 3). However, it is important to highlight that progression from HFpEF to HFrEF is somewhat atypical in humans since once HFpEF is established the progression to HFrEF mostly depends on external factors affecting heart structure (e.g., myocardial infarction) or a sustained volume overload status (e.g., obesity, kidney failure) rather than the progression of the disease itself [63,64,65]. For more individualized medical attention, further characterization of each phase with other parameters besides hemodynamic and echocardiographic measurements, such as inflammatory biomarkers, are needed to improve translational correlations.

Nevertheless, this task is complicated due to complexity not only in the chronic follow up of HHD patients, but also in initially identifying them due to asymptomatic stages. This makes ischemic HF easier to follow due to the evident initial ischemic event. Furthermore, post-MI patients require hospitalization, which leads to the wide availability of laboratory and imaging studies from baseline and during follow up. This explains why most clinical studies assessing inflammatory biomarkers, such as leukocytosis, lymphocytosis, monocytosis, and neutrophilia, and other cytokine measurements are done in ischemic HF patients. However, even though ischemic heart disease is currently the principal etiology of HF [66,67,68], changes in the prevalence of obesity, HTN, and an elderly population could alter this proportion in the future etiology of HF. Table 3 briefly highlights some important clinical correlations between non-ischemic HF patients and the murine PO-HF model. It can be observed that every stage has almost identical features, but the ADP is characterized by an acute reduction in LVEF.

As we show in Table 3, one of the biggest challenges in comparing HDD vs. PO-HF murine models is the availability of data, especially peripheral vs. local values, in which the former are either easier to measure in humans, whereas the latter is more available and specific in murine models. However, there are some similarities that are worth highlighting. First of all, the progression of the LV geometry is very similar, characterized by a period of compensatory hypertrophy before dilation. Once the left ventricle becomes dysfunctional, the pro-inflammatory cytokine, IL6, in serum, as well as the myocardial stress molecule, BNP, expression, are present and sustained through the establishment of HF [50,69,70]. Moreover, there is clinical evidence that regardless of HF etiology, IL6 correlates with severity of CHF, as well as with other parameters, such as LVEF and ventricular dysfunction, the latter probably more associated to local IL6 production rather than peripheral production [69]. Peripheral monocytes appear to be elevated in the early stages of diastolic dysfunction, and are absent in the stage of systolic dysfunction, further suggesting the transition to a cellular immune response in the chronic stages of HF. The latter is further supported by local findings of both mice and human in which T cells are infiltrated in the myocardium as well as an increased expression of adhesion molecules favoring this process. The balance of collagen synthesis vs. degradation is also crucial in the progression of the disease. In human HHD, the peripheral ratio of MMPs/TIMP1 indicates progression to HF, suggesting that tissue remodeling could be a dynamic process in the continuum of transition from asymptomatic diastolic dysfunction to symptomatic diastolic and then systolic dysfunction as it can be observed in the inversion of the ratio in stage IV of HDD [71,72,73]. In mice, even though the expression of TGFβ continues to be expressed, the time of most fibrotic tissue is at the compensatory phase, so it can be inferred that MMP´s activity increases after compensation, leading to dysfunction and eventually dilation, as well as in humans [38]. Furthermore, several studies in mice manipulating the /TIMP1 ratio, have obtained beneficial results in cardiac remodeling [74]. Nevertheless, the inflammatory response in human HDD is not completely understood in order to properly match it with the pathogenesis of the PO-HF murine model.

### 5.2. Murine Pressure Overload Heart Failure Model and Clinical Pressure Overload Heart Failure

One of the most recent studies involving only HHD patients was done by Glezeva et al. [56]. The main finding of their study was that healthy monocytes exposed to HFpEF patients’ serum acquired M2 features; although they were not non-inflammatory macrophages, they had prominent pro-fibrotic activity. Also, circulating IL6 was associated with asymptomatic LV dysfunction (LVD), which was found to be the key discriminating circulating factor in only asymptomatic HTN patients, which could suggest transition from stages. Moreover, peripheral monocytosis was associated with asymptomatic left ventricular diastolic dysfunction (LVDD) and HFpEF patients. Among these, the classical monocyte subset was associated with asymptomatic LVDD, and both classical and alternative monocytes were associated with HFpEF. Even though it is not fully confirmed, this could mean parallel progression of the monocyte subsets from classical to alternative along the pathogenesis of HHD. One limitation of this study was that all three groups were independent due to the previously mentioned logistic difficulties in early identification and long-term follow up of HHD patients. Importantly, most of the clinical studies on monocytes are focused on monocyte-derived cytokines rather than their number or proportions of subsets [76], or they are based on post-MI populations [76], which highlights the relevance of the work by Glezeva et al. [56].

Regarding the lymphocyte population, there is evidence that shows that indistinct from the etiology, HF patients tend to have reduced numbers of lymphocytes during their clinical course [76]. However, the clinical explanation for this lymphopenic phenomena is still unknown, but it has been suggested that it is related to cytokine-mediated apoptosis or splenic congestion [76]. Moreover, Nevers et al. [47] found that isolated T-cells from HF patients (NYHA Class III-IV) of non-ischemic etiology adhered to activated endothelial cells in higher numbers than those from non-HF patients. This was associated with increased T-cell infiltration in end-stage non-ischemic HF myocardium biopsies [47]. These results in non-ischemic HF patients might suggest increased lymphocyte infiltration as a likely mechanism to contribute to the paradoxical lymphopenic status, which interestingly correlates with the increased expression of leukocyte adhesion molecules in other studies with the murine PO-HF model at the HF establishment stage.

Another series of studies demonstrated that lymphopenia is associated with increased all-cause 3 year mortality and transplant-free survival in a mixed HF etiology population [77,78,79,80]. Among those studies, Acanfora et al. [80] showed that lymphocyte count was correlated to neither ischemic nor hypertensive etiology [80]. Moreover, two studies demonstrated that lymphocyte count was not associated with HF incidence during a long follow-up period among the EPIC and ARIC cohort patients [81,82], but, apparently, it does have a prognostic role in HF-diagnosed patients. This further confirmed the low association between lymphocytosis with HF etiology, but more specific correlations with prognosis of HF patients.

Regarding biomarkers, two retrospective reviews of post-hoc analyses assessed a mixed population of ischemic and non-ischemic HF patients. In summary, they found that HFrEF was associated with elevated cardiac stretch (e.g., BNP, troponin) and angiogenesis biomarkers, whereas HFpEF was associated with inflammatory (e.g., CRP, IL6, pentraxin-3) endothelial function and remodeling biomarkers (e.g., galectin-3, osteopontin). Limitations from these studies were retrospective biases with one-point measures in time and the heterogeneity of the populations [83]. However, given that galectin-3 is secreted by activated macrophages, it is important to highlight that levels of this protein have been related to be an independent marker of outcome in HF patients, with stronger predictive value in HFpEF [84], and among acute HF when combined with BNP levels [85].

A recent analysis of chronic HF prognostic scores compared their performance in a heterogeneous ambulatory HF population. They were able to demonstrate significant differences in their accuracy and the few advantages of the Meta-Analysis Global Group in Chronic Heart Failure (MAGGIC) risk model over others, specifically in predicting 1 year survival. Nevertheless, the authors highlighted that investigators are reluctant to use these score models because of their poor clinical significance for individual patients [86]. It is important to mention that none of these risk models, but Seattle Heart Failure Model (SHFM, consider any inflammatory parameters, such as leukocytes populations or circulating cytokines [86,87].

Furthermore, in an attempt to improve prognosis by giving a more individualized treatment for HF, clinical prognostic biomarkers that have been identified in HF patients have all demonstrated limited clinical applicability and significance. Thus, patient stratification by a single biomarker approach, such as TNFα cytokine, has yet to be achieved [1,2,3,4,5]. Nevertheless, a multiple biomarker approach (e.g., inflammation, stress, and extracellular matrix biomarkers) could be a plausible strategy that might help in further stratification and a more precise characterization of HF patients [5], hence improving prognosis through more individualized treatment.

## 6. Conclusions

Since the role of inflammation in HF was first described, many advances have been made in a better understanding of HF, with the ultimate goal of delivering a more efficient, tailored therapy to HF patients. Nevertheless, we have reached a therapeutic plateau in which HF is an end-stage disease with high mortality. However, in contrast to previous clinical approaches that focused on a single responsible inflammatory agent in HF progression, the newest advances in immunology have made it possible to recognize the multiple and complex interactions between the different arms of the immune system. In order to finally solve the inflammatory puzzle, research relies on HF murine models.

In this review, we described several aspects of the different phases of the PO-HF model followed by many different approaches to modify the inflammatory response and consequently HF progression. From these works, it can be concluded that different approaches at different points in time can have either similar or no effect at all. This is related to the complex interactions between the immune system and the continuously changing inflammation. Thus, there is still more to be discovered from these interactions in the progression of PO-HF.

As far as we know, this is the first work to compare and establish correlations between the different phases of the murine model of PO-HF and clinical HHD progression to HF. Given the similarities described in this review, we hope it will further help researchers and clinicians to understand PO-HF pathophysiology better and, hence, lead to better translational capabilities. Therefore, in the current quest for a more tailored therapy for HF, this disease should be considered based not only on its clinical etiology, but also in respect to the time frame (i.e., inflammatory profile) of the patient.

## Figures and Tables

**Table 1:** Comparison between PO-HF murine models.

Characteristic	TAC	ATII-Infusion
LV structure	Concentric → dilated	Concentric → dilated
Systolic function	Preserved → reduced	Preserved* → reduced*
Diastolic function	Dfxn	Dfxn
Cardiomyocyte hypertrophy	Yes	Yes
Myocardial fibrosis	Yes	Yes
Increased BNP expression	Yes	Yes
Can it be used as a HFpEF model?	No	Yes

It is evident that both models share almost all parameters. *dose and time-dependent; →: Indicates progression; Dfxn: Dysfunctional. TAC: Transverse aortic constriction; ATII: Angiotensin II; LV: Left ventricular; BP: Blood pressure; BNP: Brain natriuretic peptide; HFpEF: Heart failure preserved ejection fraction [20,21,25,26,27].

**Table 2:** Detailed description of known parameters in PO-HF.

Phase	1. ADP	2. CP	3. DP-HF		4. CHF	
Time	3rd day	1st week	2nd week	4th week	6th week	8th week
**Hemodynamic & Structural parameters**						
LVEF [40,50,51]	↓	↔	↔	↓		↓+
FS [38,47,49]	↔	↔	↓	↓	↓	
EDV [40]	↔	↔	↔	↑	↑	↑+
BP [50]		↑	↑	↑		
LV hypertrophic response [38]		concentric		eccentric	eccentric	
Fibrotic response [38,48]	↑	↑+		↑	↑	↑
RV hypertrophic response [40]						↑
**Immune cell infiltration**						
CD45^+^ [38,40]	↔	↑		↔/↑		
CD3^+^ [47,49]	↔	↑	↑	↑	↑	↑
CD3^+^, CD4^+^ [47,49]				↑	↑	↑
CD3^+^, CD8^+^ [48,49]				↑	↔	
Treg [48]						↑
M [38,40]	↔	↑	↔	↔		
M1 [48]	↔	↑		↔		
M2 [48]	↔	↑		↔		
Gr1^+^ [47]	↔		↔			
Classical DCs [40]	↔	↑		↔		↑
**Peripheral circulating immune cells**						
Mon Ly6C^hi^ [40]	↔	↑		↔		
Mon Ly6C^lo^ [40]	↔	↔		↔		
Cytokines:						
BAFF (B cell activating factor) [50]				↑		
IL-1β, IFN-ɣ, TNFα, IL-6, IL-10 [50]				↔		
**Mediastinic Lymph Node**						
CD3^+^ [47]		↑		↑	↑	↑
CD3_+_, CD4^+^ [47]		↑		↑	↑	
CD3^+^, CD8^+^ [47]		↑			↔	
**Gene Expression**						
BNP [48]		↑		↑		
TGF-β [38]		↑		↑		
Chemokines:						
CCL4, CCL5, CXCL11 [51]		↑		↔		
MCP-1(CCL2) [38,51]		↑		↔		
CCL7, CCL12 [48]		↑				
CXCL10, CX3CL1, CXCL16, CCL17 [49]					↑	
CD3e [51]				↑		
Cytokines:						
TNFα, IL-6, IFN-ɣ [47,48,49,50,51]		↑		↑		
IL-1β, IL-8, BAFF [47,50,51]				↑		
IL-4 [48,51]		↓		↑		
IL-10 [48,50]		↔		↓		
RORɣt [47]				↑		
Foxp3 [47,51]				↔		
Adhesion molecules						
VCAM-1, E-Sel, ICAM-1 [47]				↑		
**Heart tissue immunostaining**						
IgG1, IgG2, IgG4 [50]				↓		
IgG3 [50]				↑		
BAX [50]				↑		
Anti-ssDNA [50]				↑		

ADP: Acute decompensatory phase; CP: Compensatory phase; DP-HF: Decompensatory phase and heart failure establishment; CHF: Chronic and congestive heart failure. ↑: Increased; ↓: Decreased; ↔: Without changes; +: Most increased or decreased value of same parameter along timeline. LVEF: left ventricular ejection fraction; FS: fraction shortening; EDV: end diastolic volume; BP: blood pressure; LV: left ventricular; RV: right ventricular; CD45^+^: transmembrane protein tyrosine phosphatase located on most haematopoietic cells; CD3^+^: T cells; CD3^+^, CD4^+^: T helper cells; CD3^+^, CD8^+^: T cytotoxic cells; Treg: regulatory T cells; M: macrophages; M1: pro-inflammatory macrophage; M2: anti-inflammatory macrophage; BNP: brain natriuretic peptide; TGF-β: transforming growth factor beta; CCL: chemokine (cysteine-cystein) ligands; CXCL: chemokine (C-X-C motif) ligand; MCP-1: monocyte chemoattractant protein-1; CD3e: CD3 epsilon chain; RORɣt: RAR-related orphan nuclear receptor ɣt; Foxp3: forkhead box P3; VCAM-1: vascular cell adhesion molecule 1 ; E-Sel: E-selectin, ICAM-1: intercellular adhesion molecule 1; IgG: immunoglobulin G; BAX: proapoptotic protein; ssDNA: single stranded DNA.

**Table 3:** Comparison between human and murine progression of PO-HF

Human Progression of HDD [62,63]							Murine Progression of PO-HF [38,40]						
Degree	Dfxn	LV	EF	Peripheral		↑Local markers	Phase	Dfxn	LV	EF	Peripheral		↑ Local markers
				↑Cytokines	↑ Cells						↑Cytokines	↑ Cells	
I	D	P	P	IL6, CRP, MMP9, BNP [56,69,71,75]	Mon [56]		ADP	P	Preserved	R			
II	D	C-LVH	P				CP	P	C-LVH	P		Mon [40]	T cell, macrophages, DCs [40,47,48,49] BNP, TGFB, MCP1, CCLs, TNFa, IL6, IFN-ɣ [38,47,48,49,52]
III	D	C-LVH	P	TNFα, MCP1, IL6, IL12, IL8, MMP9, BNP [56,71]	Mon [56]	Col-I, Col-3, Col-1/Col-3 TGF-β, VCAM-1, TIMP1, MMP2, ↓MMP1, Lukocytes, T-cells, CD11a+ cells [72]	DP-HF	S	Dilation	P	BAFF [50]		T cell [47,48,49] BNP, TGFB, MCP1, TNFa, IL6, IL8, IFN-ɣ. IL1B, IL4, ↓IL10, VCAM-1, ICAM-1, E-Sel [38,47,48,49,50,51]
IV	S	Dilation	R	TNF α, MMP1/TIMP1, BNP [16,73]		T cells, TNFα, ↓TNFR [16,47]	CHF	S	Dilation	R			T cell, DCs [40,47,49]

ADP: Acute decompensatory phase; C-LVH: Concentric left ventricle hypertrophy; CP: Compensatory phase; DP-HF: Decompensatory phase and heart failure establishment; CHF: Chronic and congestive heart failure. Dfxn: Ventricle dysfunction; D: Diastolic; S: Systolic; P: Preserved; R: Reduced; EF: Ejection fraction. ↑: increased; ↓: decreased.

## Abbreviations

ADP	Acute decompensated phase
ATII	Angiotensin II
BNP	Brain natriuretic peptide
BP	Blood pressure
CHF	Congestive and end-stage heart failure
CMP	Cardiomyopathy
CP	Compensated phase
DCs	Dendritic cell
DP-HF	Decompensated phase and heart failure establishment
Dsfx	Dysfunction
EDV	End-diastolic volume
EF	Ejection fraction
E-Sel	E-selectin
HFmrEF	Heart failure with moderately reduced ejection fraction
HFpEF	Heart failure with preserved ejection fraction
HFrEF	Heart failure with reduced ejection fraction
HTN	Hypertension
ICAM-1	Intercellular adhesion molecule 1
IFN-ɣ	Interferon-ɣ
IgG	Immunoglobulin G
IL	Interleukin
LVEF	Left ventricle ejection fraction
M1	Pro-inflammatory macrophage
M2	Anti-inflammatory macrophage
MAP	Mean arterial pressure
MCP1	Monocyte chemoattractant protein-1
MLN	Mediastinal lymph node
Mon	Monocyte
MnPs	Mononuclear phagocytes
NYHA	New York Heart Association
PMN	Polymorphonuclear neutrophils
PO	Pressure overload
PO-HF	Pressure overload heart failure
RV	Right ventricle
TAC	Transverse aortic constriction
TGFβ	Transforming growth factor-β
TNFα	Tumor necrosis factor-α
Treg	T regulatory cell
VCAM-1	Vascular cell adhesion molecule 1
